# Post-traumatic syringomyelia

**DOI:** 10.4103/0019-5413.37006

**Published:** 2007

**Authors:** Amit Agrawal, M Shantharam Shetty, Lekha Pandit, Lathika Shetty, U Srikrishna

**Affiliations:** Department of Surgery, Datta Meghe Institute of Medical Sciences, Sawangi (Meghe), Wardha, India; * Department of Orthopedics, K.S. Hegde Medical Academy, Mangalore, India; ** Department of Neurology, K.S. Hegde Medical Academy, Mangalore, India; *** Department of Radiology, K.S. Hegde Medical Academy, Mangalore, India

**Keywords:** Post-traumatic syringomyelia, spinal cord, syringomyelia, trauma

## Abstract

Progressive post-traumatic cystic syringomyelia is an uncommon and increasingly recognized cause of morbidity following spinal cord injury. We hereby report a 35-year-old gentleman who sustained wedge compression fracture of L-1 vertebral body 15 years back and had complete paraplegia with bowel/bladder involvement. The neurological deficit recovered with minimal residual motor deficits and erectile dysfunction. He presented now with increasing neurological deficits associated with pain and paresthesia. The MRI spine showed a syrinx extending from the site of injury up to the medulla. He underwent a syringo-peritoneal shunt and at followup his pain and motor functions had improved but erectile dysfunction was persisting.

Progressive post-traumatic cystic syringomyelia is an uncommon but potentially clinically serious complication of spinal cord injury.^1^ Post-traumatic delayed development of syringomyelia, once thought to occur in only 1-4% of cases, is now increasingly recognized as a major cause of morbidity.^1^ Progressive signs and symptoms can develop as early as three months after spinal cord trauma or can occur as late sequelae as late as 32 years after spinal cord injury.^2^–^4^ We report a case of post-traumatic syringomyelia and review the relevant literature.

## CASE REPORT

**Figure 1:**
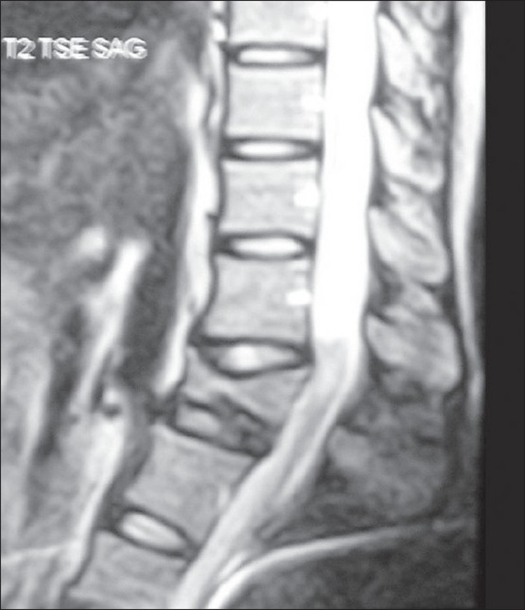
Mid sagittal T2-weighted image of MRI of dorso-lumbar spine shows the post-traumatic khyphosis at the L1 level with syringomyelia

**Figure 2:**
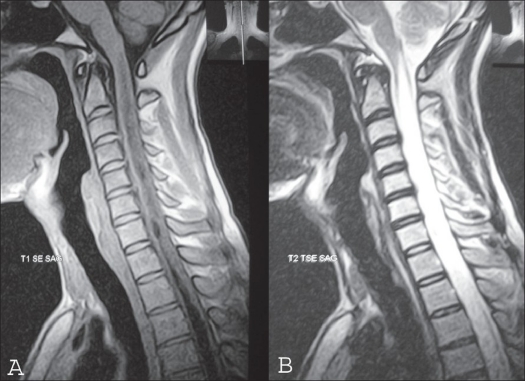
Mid sagittal T1-weighted image of MRI cervical spine (A) and T2 WI (B) shows syringomyelic cavity extending up to the medulla

A 35-year-old gentleman sustained wedge compression fracture of L-1 vertebral body 15 years back following a fall from height. At that time the patient had complete paraplegia with bowel/bladder involvement and was managed conservatively. He made a gradual but complete bowel/bladder recovery and had minimal motor dysfunction in lower limbs. Now he presented with difficulty in holding objects in the right hand, of one month duration, decreased sensation for cold and hot water and associated burning pain and paraesthesias. For the last 15 days the patient had noticed the same complaints in the left upper limb also. He also noticed weakness of the lower limbs. There were no bowel/bladder disturbances but he had erectile dysfunction. His general and systemic examination was unremarkable. Higher mental functions were normal. Motor examination revealed wasting of small muscles of both hands and thinning of both lower limbs. Power was Grade 3/5 in the upper limbs and 4/5 in the lower limbs. All deep tendon reflexes were sluggish. Pain and temperature sensations were impaired in both upper limbs and position and vibration sense were also impaired in the lower limbs. Blood investigations were normal. X-ray cervical spine was normal. X-ray lumbar spine showed old healed fracture of L1 body. The MRI spine revealed a syringomyelia extending from the site of vertebral injury at L1 [Figure 1] to the level of the medulla [Figures 2]. The patient underwent cystoperitoneal shunt. Surgery was performed in lateral position. D11 and 12 laminectomy was performed. Shunt was placed at the D11-12 level above the level of conus and the shunt tip was introduced at the most prominent and thinned out part of the cord and advanced approximately 2-3 cm into the cystic cavity. Pial stitches were taken to hold the shunt in place. At 14 months followup his pain and paresthesias were reduced, power in the upper limbs improved to Grade 4/5 but erectile dysfunction was persisting.

## DISCUSSION

The pathogenesis of syringomyelia formation is unclear, although several theories have been proposed. Ascending and descending post-traumatic myelopathy is thought to begin at the time of injury or soon thereafter and may involve mechanisms such as the release of destructive enzymes, free radical products or other toxic agents.^5^,^6^ Central or dorso-central cores of necrotic tissue may extend for several spinal segments from the level of injury and may liquefy to form a cavity.^1^,^5^ This probably accounts for the apparent continuum of non-cystic to cystic myelopathy. The rate of progression of the pathologic process in individual cases is highly variable.^7^ Several theories are postulated to explain post-traumatic syrinx formation and include rupture and coalescence of microcysts.^8^ Microcyst formation in contused, compressed and transected spinal cords results in part from the egress of fluid from damaged axons.^9^,^10^ Various mechanisms including resorption of blood and necrotic tissue, lysomal autodigestion, secretion of cystic fluid by the developing cyst lining, infarction have been proposed. The post-traumatic reactive ependymal proliferation causing segmental closure of the central canal with resulting local distension and passage of cerebrospinal fluid (CSF) into the injured central cord via enlarged perivascular spaces has also been suggested.^1^,^2^,^10^ The formation of arachnoid adhesions may further alter the CSF dynamics so that abnormal entry of CSF into the spinal cord via Virchow-Robin spaces results in progressive syringomyelia.^7^ As in the present case post-traumatic syringomyelia most commonly extends superiorly from the injury site; less frequently, it may also have an inferior component.^11^ The mechanism involved in the progressive enlargement of post-traumatic syrinx cavities remains uncertain and includes the presence of scarring and adhesions within the subarachnoid space, alterations in CSF flow dynamics and fluid turbulence within the syrinx cavity itself.^12^,^13^ As in the present case, the first clinical suggestion of advancement of a post-traumatic syringomyelia is usually the onset of ascending signs, severe pain or the development of new symptoms (i.e. sensory loss to a fully developed anesthesia with motor weakness of an extremity) after an apparently stable period and warrants further investigations.^10^,^14^ On MRI, hyperintense T2 signals within the spinal cord parenchyma adjacent to syringomyelia may correlate with both edema formation and myelin degeneration with or without gliosis. Such MR signal change is not typically observed distant from the site of focal cord trauma; permanent parenchymal signal alteration is usually only seen immediately surrounding the primary focus of cord trauma.^12^ Resolution of these abnormal parenchymal MR signal intensities and improvement in advancing signs and symptoms after surgical CSF diversion suggests that this may be due to fluid escaping from the syrinx or edema caused by as yet undefined pathologic alterations of the spinal cord adjacent to the enlarging syrinx.^12^ Ascending/advancing clinical signs and symptoms, the rapidity of progression, the extent of syringomyelia, the diameter of the syrinx, the instability factor of the primary injury, the extent of cord injury/compression by the primary injury, the neurological status and age of the patient, all are equally important and need to be evaluated while the patient is being considered for surgery as CSF diversion in these large cysts may halt the progression of the myelopathy and reverse the clinical deterioration in many cases.^11^,^12^

## CONCLUSION

In a patient with history of previous spinal injury and new onset of neurological deficits, we should be aware of the possibility of post-traumatic syringomyelia, and subsequent surgical intervention in these patients may halt the progression of the myelopathy and reverse the clinical deterioration.
